# Comprehensive Identification of the Chemical Components in the Classical Prescription Shashen Maidong Decoction Based on UPLC-Q-Orbitrap MS and Molecular Networking

**DOI:** 10.3390/ph19071044

**Published:** 2026-07-05

**Authors:** Kun Zhang, Weide Xing, Qiang Wang, Haiyan He, Xingliang Xie, Dingkun Zhang, Yue Qi, Ming Yang

**Affiliations:** 1 School of Pharmacy, Chengdu University of Traditional Chinese Medicine, Chengdu 611137, China; zk@puhuazy.com (K.Z.); xingweide@stu.cdutcm.edu.cn (W.X.); wq@puhuazy.com (Q.W.); hehaiyan@stu.cdutcm.edu.cn (H.H.); zhangdingkun@cdutcm.edu.cn (D.Z.); 2 Sichuan Pu Hua TCM Technology Co., Ltd., Chengdu 611100, China; 3 College of Modern Traditional Chinese Medicine Industry/Tianfu Traditional Chinese Medicine Innovation Port, Chengdu University of Traditional Chinese Medicine, Chengdu 611930, China; xiexingliang@cdutcm.edu.cn; 4 College of Chemistry, Sichuan University, Chengdu 610064, China; 5 National Key Laboratory for Modern Chinese Medicine Creation Based on Classical Prescriptions, Jiangxi University of Traditional Chinese Medicine, Nanchang 330004, China

**Keywords:** Shashen Maidong Decoction, ultra-performance liquid chromatography-quadrupole-electrostatic field orbitrap high-resolution mass spectrometry, molecular networking

## Abstract

**Background/Objectives:** Shashen Maidong Decoction (SMD) has a long history of use within the traditional Chinese medicine (TCM) system and is currently employed in modern clinical practice for the treatment of various diseases. The characterization of the chemical constituents of TCM drugs is a prerequisite and foundation for research into bioactive compounds and quality control. However, no study has yet undertaken a comprehensive identification of its chemical constituents. Therefore, it is necessary to establish suitable analytical methods to comprehensively and systematically characterize the chemical constituents of SMD. **Methods:** Ultra-performance liquid chromatography-quadrupole-electrostatic field orbitrap high-resolution mass spectrometry (UHPLC-Q Exactive orbitrap HRMS) and the Global Natural Products Social Molecular Networking (GNPS) technology were employed. The chemical constituents in SMD were systematically identified by comparing mass spectrometry data with reference standards, databases and relevant literature, and by analyzing mass spectrometry fragmentation patterns. **Results:** A total of 86 compounds were identified in SMD, including 27 flavonoids, 2 homoisoflavonoids, 34 organic acids, 2 alkaloids, 4 amino acids, 5 saccharides, 3 triterpenes and 9 other constituents. **Conclusions:** This study represents the first relatively comprehensive and systematic characterization of the chemical constituents in SMD, enriching modern understanding of SMD and laying the foundation for the identification of bioactive compounds, the elucidation of mechanisms of action, and further development and utilization.

## 1. Introduction

Classical prescriptions of Traditional Chinese Medicine (TCM) consist of combinations of various natural products designed to prevent and treat diseases. Their unique therapeutic effects and mechanisms of action have been validated by long-term clinical practice among TCM practitioners. Classic formulas from TCM represent the essence of the theoretical framework of traditional Chinese medicine, boasting a rich historical heritage and proven clinical efficacy [1,2]. Shashen Maidong Decoction (SMD) has a long history of use in China and has demonstrated reliable therapeutic effects [3]. The formula is derived from “Wen Bing Tiao Bian” by the renowned Qing Dynasty physician Jutong Wu. The text states: “For dryness injuring the yin aspect of the lung and stomach, presenting with fever or cough, SMD is the primary treatment.” Traditionally, it has been primarily used to treat the TCM syndrome pattern of “dryness injuring the yin of the lung and stomach”—a respiratory disorder characterized by persistent cough with scant sputum, and dryness and thirst in the mouth and throat, resulting from dry pathogenic factors or internal heat depleting the yin and body fluids of the lung and stomach. Modern research indicates that, in addition to its use in respiratory diseases [4], such as pneumonia [5,6], chronic bronchitis [7] and lung cancer [8,9]. SMD is also applied in the clinical treatment of a wide range of conditions, including digestive system disorders, ear, nose, and throat disorders and endocrine disorders [10].

The pharmacologically active constituents of TCM herbs and their formulations serve as a crucial link between chemical components and clinical efficacy. They are a core element in ensuring the efficacy and safety of clinical use, and also a central aspect of research into the modernization of TCM. SMD comprises seven herbs including Adenophorae Radix (chinese name: shashen, the root of *Adenophora tetraphylla* (Thunb.) Fisch. or *Adenophora stricta* Miq.), Ophiopogonis Radix (chinese name: maidong, the root of *Ophiopogon japonicus* (L. f.) Ker-Gawl.), Polygonati Odorati Rhizoma (chinese name: yuzhu, the root of *Polygonatum odoratum* (Mill.) Druce), Trichosanthis Radix (chinese name: tianhuafen, root of *Trichosanthis kirilowii* Maxim. or *Trichosanthes rosthornii* Harms), Lablab Semen Album (chinese name: baibiandou, the seed of *Dolichos lablab* L.), Mori Folium (chinese name: sangye, the leaf of *Morus alba* L.) and Glycyrrhizae Radix et Rhizoma (Chinese name: gancao, the root of *Glycyrrhiza uralensis* Fisch., *Glycyrrhiza inflata* Bat. or *Glycyrrhiza glabra* L.). Although SMD is widely used and studied in clinical practice, a comprehensive understanding of its chemical profile remains lacking. Clarifying the chemical constituents of this formula is of great significance for elucidating its pharmacological basis, comprehensively evaluating and controlling its quality, and thereby guiding clinical application. Therefore, it is necessary to establish systematic analytical and identification methods to enable the rapid identification and accurate determination of the various constituents in SMD.

Liquid chromatography-mass spectrometry (LC-MS) combines the efficient separation capabilities of liquid chromatography (LC) for complex samples with the high sensitivity and powerful qualitative capabilities of mass spectrometry (MS), enabling effective differentiation of compounds with similar molecular weights [11,12]. Thus, it has become a core method for the rapid characterization and identification of chemical constituents in TCM drugs. In particular, high-resolution mass spectrometry can provide precise mass information on chemical constituents, which is used to accurately deduce their chemical formulas and thereby elucidate their structures [13,14]. Quadrupole-Orbitrap high-resolution mass spectrometry (Q-Orbitrap MS) offers the significant advantages of high resolution, high sensitivity and high stability. By collecting first- and second-order mass spectrometry data for target compounds and comparing this with reference standards and mass spectrometry databases, it is possible to rapidly identify target compounds. Currently, this technology has been widely applied in the chemical identification of TCM herbs and their compound formulations [15,16]. In recent years, molecular networking (MN) on the Global Natural Products Social Molecular Networking (GNPS) platform has emerged as a novel data analysis approach and has gradually become a key research strategy for the discovery and identification of naturally occurring bioactive molecules. MN is a technique that maps the spectral structural space by comparing the similarity of secondary fragments of compounds, and clusters compounds into groups when the similarity exceeds a set threshold [17,18]. At present, LC-MS/MS-based molecular networking is not only widely applied to natural products such as microorganisms [19], fungi [20], marine organisms [21] and plants [22], but its application in the study of single TCMs and TCM herbal formulae is also showing a rapidly growing trend [23,24].

To date, no study has reported a comprehensive identification of the chemical constituents in SMD, which has, to some extent, hindered a deeper understanding of this classic TCM formula. Therefore, this study employed UHPLC-Q Exactive Orbitrap MS combined with MN approach to systematically analyze the major chemical constituents in SMD. The aim is to provide a comprehensive overview of the major compounds of SMD and to summarize their fragmentation patterns, thereby providing more in-depth scientific data to support future research into their in vivo metabolic processes, the determination of quality parameters, and subsequent pharmacological studies, thus enhancing their scientific value.

## 2. Results

**Figure 1:**
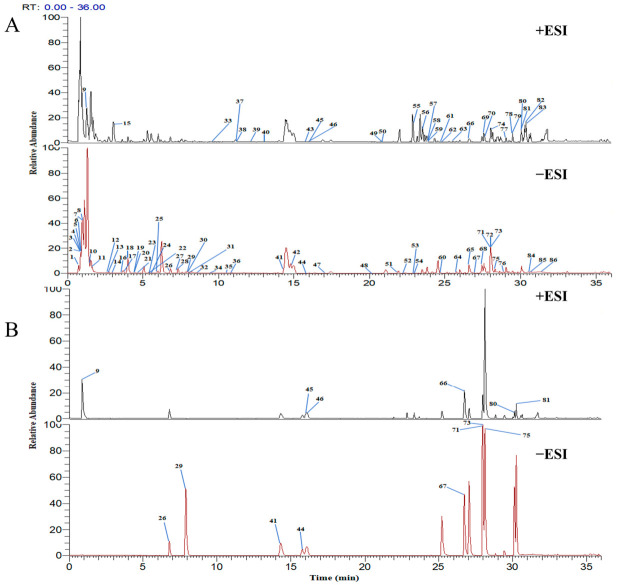
The total ion flow diagram of SMD (**A**) and the mixed standard solution (**B**) by UHPLC-Q Exactive orbitrap HRMS.

**Figure 2:**
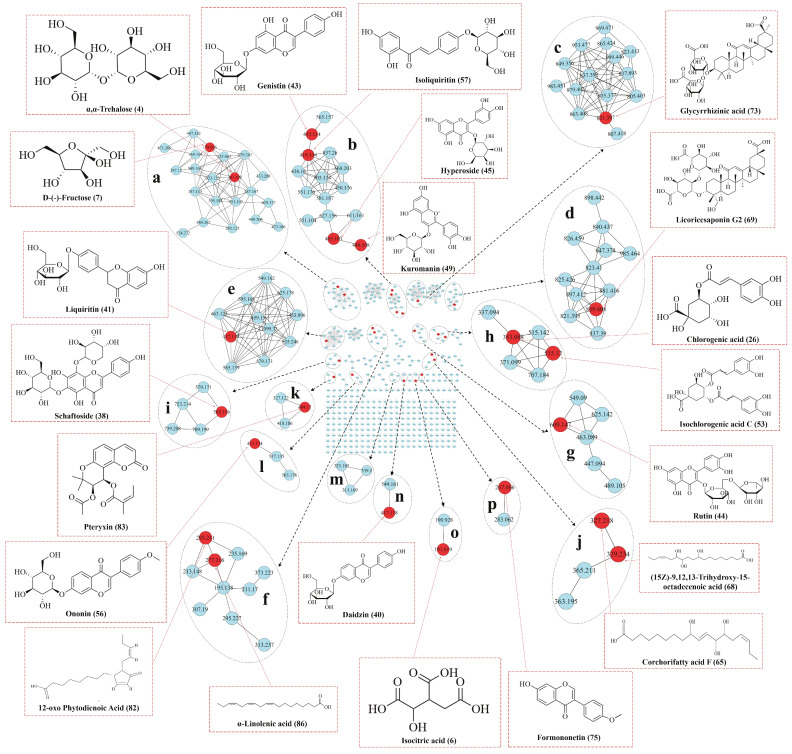
Visualization map of the molecular network of SMD. Saccharides (**a**), flavonoids (**b**,**e**,**g**,**i**,**l**,**n**,**p**), organic acids (**f**,**h**,**j**,**o**), triterpenes (**c**,**d**), coumarins (**k**), alkaloids (**m**).

**Figure 3:**
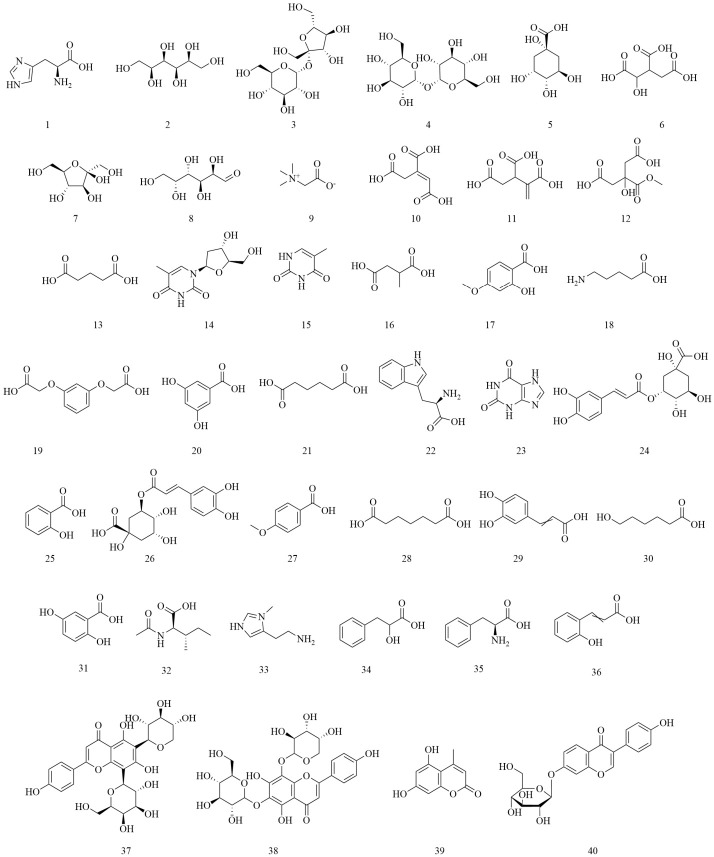

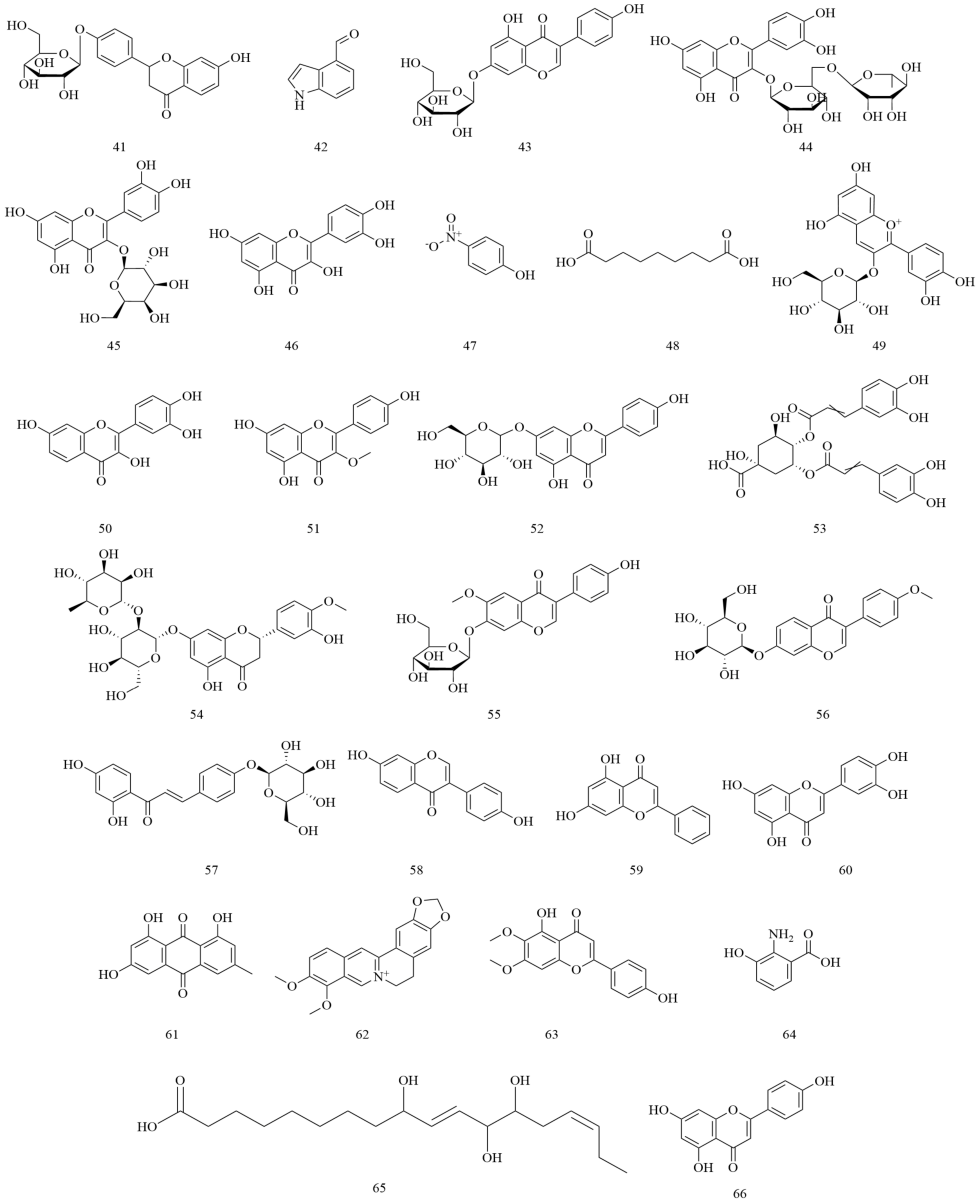

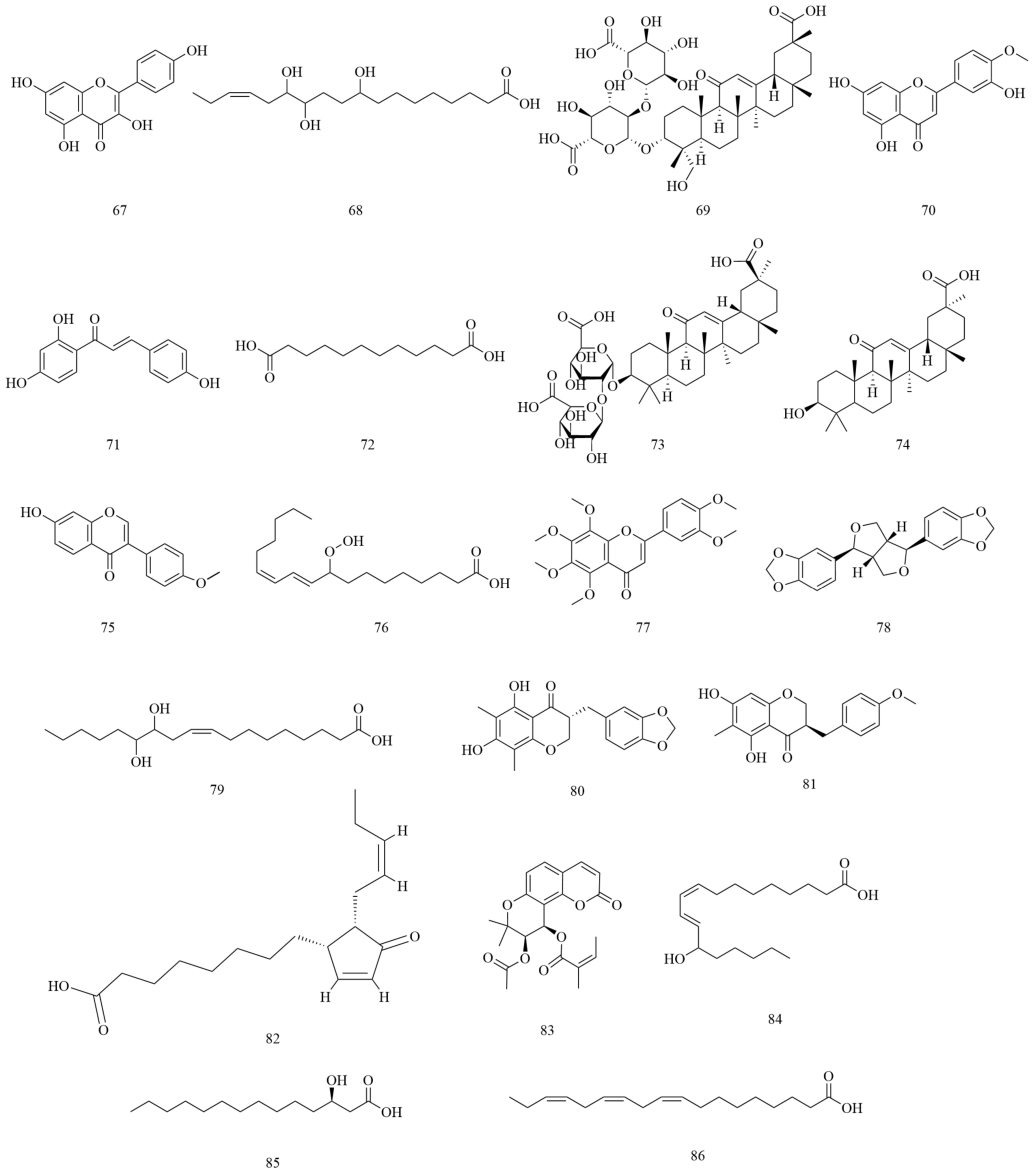
Chemical structures of characterized compounds in SMD.

In this study, by comparing retention times (RT) and mass spectrometry (MS) data using GNPS, databases and existing literature, a total of 86 compounds were definitively identified, of which 14 were also compared with reference standards. The identified compounds include 27 flavonoids, 2 homoisoflavonoids, 34 organic acids, 2 alkaloids, 4 amino acids, 5 saccharides, 3 triterpenes and 9 other constituents. The total ion flow diagram (TIC) of SMD (A) and the mixed standard solution (B) obtained in positive and negative ionization modes are shown in Figure 1 (The normalization method is “Largest peak in selected time range”). The visualization results obtained from the GNPS molecular network (MN) analysis are shown in Figure 2. As certain compounds had not been reported in the existing literature and lacked secondary mass spectrometry data for reference, these compounds were disregarded and excluded. Information on the identified compounds is presented in Table 1, and structures of each compound are shown in Figure 3.

### 2.1. Identification and Structural Analysis of Saccharides

In this study, a total of five sugar compounds were identified from SMD in both positive and negative ion modes, including Iditol (2), Sucrose (3), α,α-Trehalose (4), Fructose (7) and Galactose (8). The molecular network identified two sugar compound nodes within the molecular cluster of Saccharides (Figure 2a). Take compound **5** as an example to analyze the fragmentation patterns.

**Figure 4:**
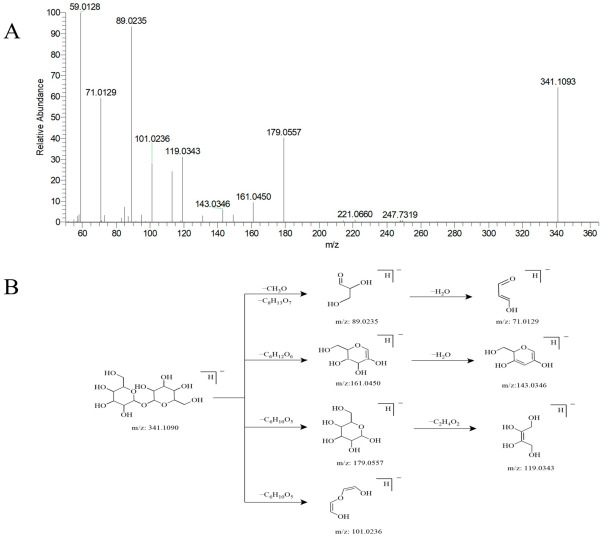
MS2 spectra (**A**) and fragmentation pathways (**B**) of α,αTrehalose.

In negative ion mode, the quasi-molecular ion peak for compound **4** is m/z: 341.1090 [M−H]^−^, with the Xcalibur software 4.0 fitting the corresponding molecular formula as C_12_H_22_O_11_. During the mass spectrometry fragmentation process, the molecular ion peak can be observed to sequentially lose CH_3_O and C_8_H_13_O_7_ to form the fragment ion peak m/z 89.0235 [M-H-CH_3_O-C_8_H_13_O_7_]^−^, and subsequently, upon the loss of H_2_O, the fragment ion m/z 71.0129 [M-H-CH_3_O-C_8_H_13_O_7_-H_2_O]^−^ was observed. In addition, the following fragment ions were also observed: m/z 101.0236 [M-H-C_4_H_8_O_4_-C_4_H_8_O_4_]^−^, m/z 161.0450 [M-H-C_6_H_12_O_6_]^−^, m/z 143.0346 [M-H-C_6_H_12_O_6_-H_2_O]^−^, m/z 179.0557 [M-H-C_6_H_10_O_5_]^−^ and m/z 119.0343 [M-H-C_6_H_10_O_5_-C_2_H_4_O_2_]^−^. Based on the exact molecular mass in negative ion mode and the characteristic fragment ion information, combined with data from the literature [28,29], the compound was identified as α,α-Trehalose. Its possible cleavage pathways are shown in Figure 4.

### 2.2. Identification and Structural Analysis of Flavonoids

Flavonoids are a class of compounds that are widely found in nature. They are distinguished by having a basic C6-C3-C6 skeleton, which is made up of two benzene rings connected by three carbon atoms. Through comparison with reference standards, analysis of secondary mass spectrometry fragmentation patterns, and comparison with literature data, a total of 27 flavonoid components were identified or inferred from the SMD. These represent one of the major classes of compounds identified by SMD and are primarily derived from Glycyrrhizae Radix and Mori Folium. Concurrently, MN analysis identified seven clusters of flavonoid compounds. The fragmentation patterns of these compounds are similar, typically involving the cleavage of glycosidic bonds, Diels–Alder (RDA) cleavage of the C-ring of the flavanone aglycone, and ion loss patterns such as CO, H_2_O and CO_2_ [87].

**Figure 5:**
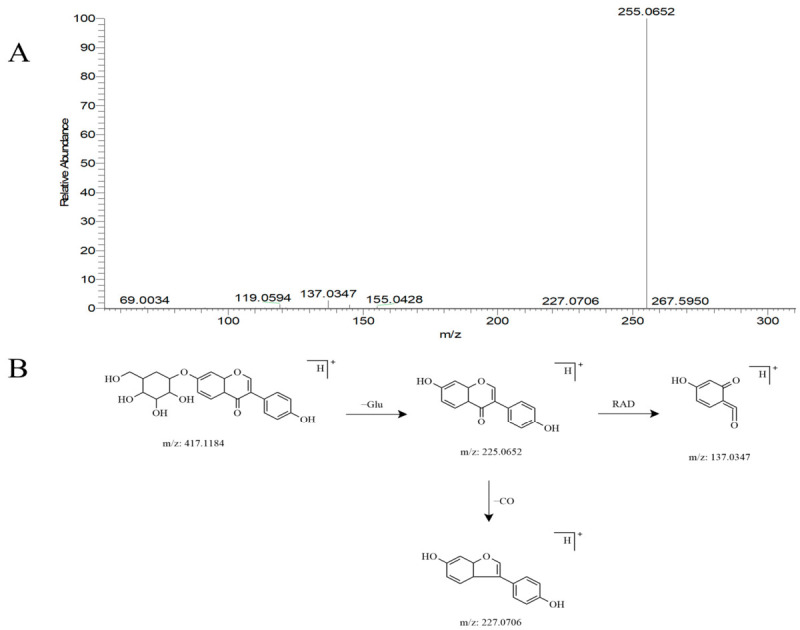
MS2 spectra (**A**) and fragmentation pathways (**B**) of daidzin.

In positive ion mode, compound **40** produces a parent ion peak at m/z 417.1184, corresponding to the ion mode [M+H]^+^. The parent ion undergoes the aforementioned splitting rule to generate the corresponding fragment ion peaks. First, through the cleavage of the sugar chain and the loss of one glucose unit (-Glu), a fragment ion of m/z 255.0665 [M+H-Glu]^+^ is formed. Subsequently, RDA cleavage or further fragmentation occurs on the C ring of this ion, resulting in the loss of CO and the generation of fragment ions m/z 137.0347 [C_7_H_4_O_3_+H]^+^, and m/z 227.0706 [M+H-Glu-CO]^+^ fragment ions. Based on the above cleavage patterns, it is inferred that compound **40** is Daidzin [60]. The possible mass spectrometry cleavage pathways are shown in Figure 5.

**Figure 6:**
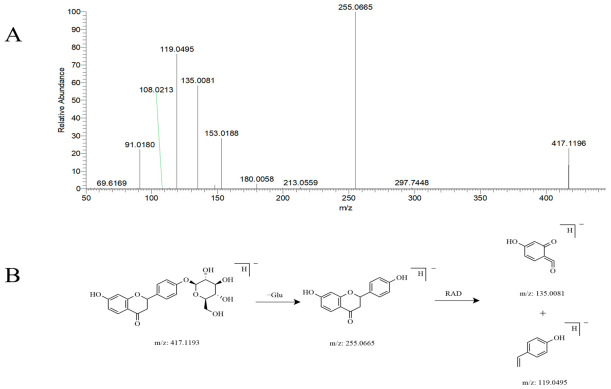
MS2 spectra (**A**) and fragmentation pathways (**B**) of liquiritin.

The precursor ion of compound **41** is m/z 417.1193 [M−H]^−^, which undergoes the same typical cleavage reaction. Based on the secondary mass spectrometry data, m/z 255.0665 can be inferred; [M-H-Glu]^−^ is produced by the loss of a glucose residue (162 Da), whilst an RDA cleavage reaction on the C ring yields the fragment ions m/z 135.0081 [C_7_H_4_O_3_-H]^−^ and m/z 119.0495 [C_8_H_8_O-H]^−^. By comparing the secondary mass spectrometry data with that of reference standards and literature reports, the compound was identified as Liquiritin [61,62], and its possible cleavage pattern is shown in Figure 6.

**Figure 7:**
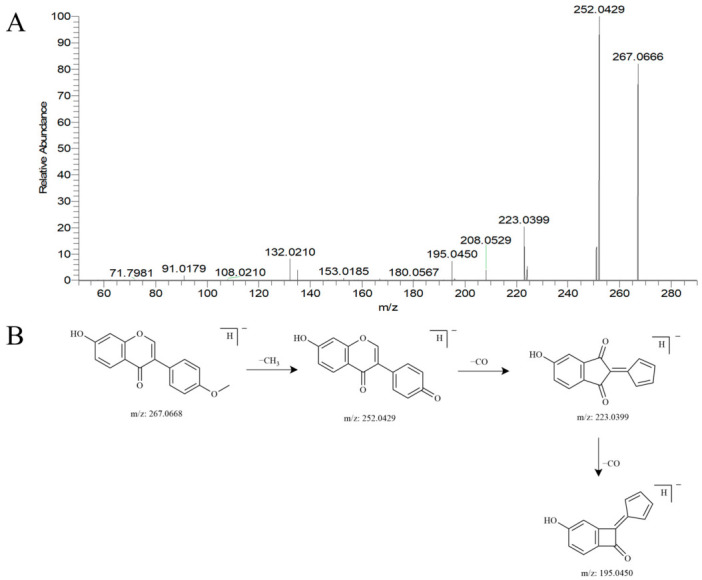
MS2 spectra (**A**) and fragmentation pathways (**B**) of formononetin.

Compound **75** has a retention time of 27.99 min, with a parent ion at m/z 267.0668 [M−H]^−^, with the software-fitted molecular formula being C_16_H_12_O_4_. Its secondary fragment ions are m/z 252.0429 [M-H-CH_3_]^−^, m/z 223.0399 [M-H-CH_3_-CO]^−^, and m/z 195.0450 [M-H-CH_3_-2CO]^−^. These are characteristic fragment ions obtained when the quasi-molecular ion first loses a CH_3_ group, followed by further cleavage. Based on database entries, reference standards and fragment information reported in the literature, the compound was identified as Formononetin [28,79]. The possible cleavage mechanism is shown in Figure 7.

Compound **60** exhibits an excimer ion peak at m/z 285.0410 [M−H]^−^. In secondary mass spectrometry, m/z 151.0034 [M-H-C_8_H_6_O_2_]^−^ and m/z 133.0288 [M-H-C_7_H_4_O_4_]^−^ fragments, resulting from RDA cleavage of the parent ion, were observed. Furthermore, the loss of CO_2_ from the C-ring of the parent ion to the characteristic fragment m/z 241.0449 [M-H-CO_2_]^−^ was observed; comparison with data reported in the literature suggests that Compound **60** is luteolin [60].

### 2.3. Identification and Structural Analysis of Homoisoflavonoids

Methylophiopogonanone A (80) and methylophiopogonanone B (81) are two homoisoflavonoids identified from SMD, both derived from Ophiopogonis Radix. Homoisoflavonoids belong to a special class of flavonoids; their structure contains one additional methylene group compared to isoflavones, formed by the attachment of a benzyl group to the C3 position of chromone or chromanone. Due to the presence of the methylene group, this position is prone to cleavage during high-energy collisions in mass spectrometry, causing the bond between the C ring and the B ring to break and thereby generating fragment ions lacking the B ring.

**Figure 8:**
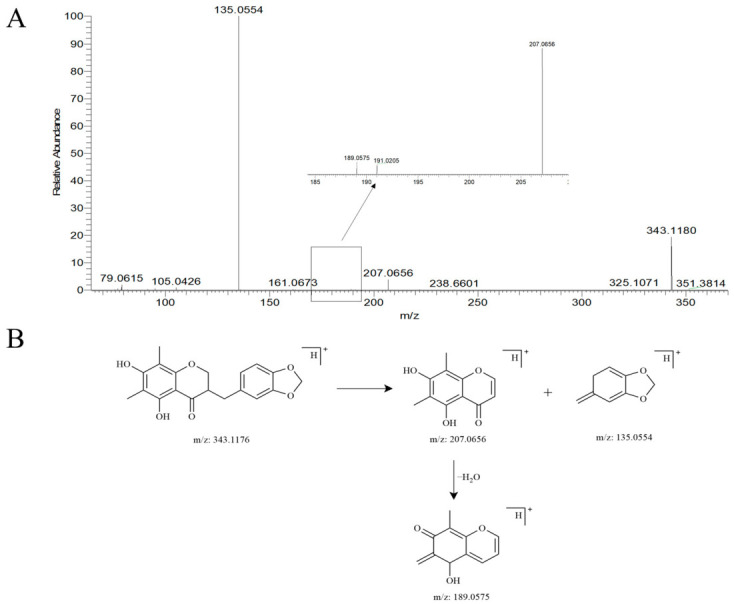
MS2 spectra (**A**) and fragmentation pathways (**B**) of methylophiopogonanone A.

In the cation mode, the protonated molecule of compound **80** was detected at m/z 343.1176 [M+H]^+^, corresponding to the molecular formula C_19_H_18_O_6_. Subsequently, the bond connecting the C ring and the B ring in this molecule cleaved, yielding 207.0653 [M+H-C_8_H_8_O_2_]^+^, and 135.0554 [M+H-C_12_H_10_O_2_]^+^. The former immediately loses a H_2_O molecule, yielding 189.05757 [M+H-C_8_H_8_O_2_-H_2_O]^+^. Following comparison with databases and reference standards, and in conjunction with the characteristic ion fragments reported in the literature [83], this compound was identified as methylophiopogonanone A. Its possible cleavage pattern and secondary mass spectrum are shown in Figure 8.

Compound **81** (m/z 327.1233, [M+H]^+^), corresponding to the molecular formula C_19_H_20_O_5_, exhibited the same fragmentation behavior during mass spectrometry: the bond connecting the C ring and the B ring was cleaved, yielding 207.0653 [M+H-C_8_H_9_O]^+^ and 121.0750 [M+H-C_11_H_11_O_4_]^+^, followed by the loss of -CH_3_ at m/z 106.0504 [M+H-C_11_H_11_O_4_-CH_3_]^+^, yielding the major fragment ion detected. Based on literature data and comparison with reference standards [83], this compound was identified as methylophiopogonanone B.

### 2.4. Identification and Structural Analysis of Organic Acids

In nature, organic acids are a class of acidic organic compounds that are commonly found in plants. In mass spectrometry, they often appear as [M−H]^−^ precursor ion peaks and, upon high-energy collisions, primarily form fragment ions such as [M-H-H_2_O]^−^ or [M-H-CO_2_]^−^. Analysis has identified 40 organic acid components in the SMD. Based on differences in carboxyl groups and linking groups, these are classified into two major categories: fatty acids and phenolic acids. The organic acids identified in this study are widely distributed across seven Chinese herbal medicines in the SMD. Within the molecular network, four clusters labeled as organic acids were identified, annotating a total of eight nodes (Figure 2f,h,j,o).

**Figure 9:**
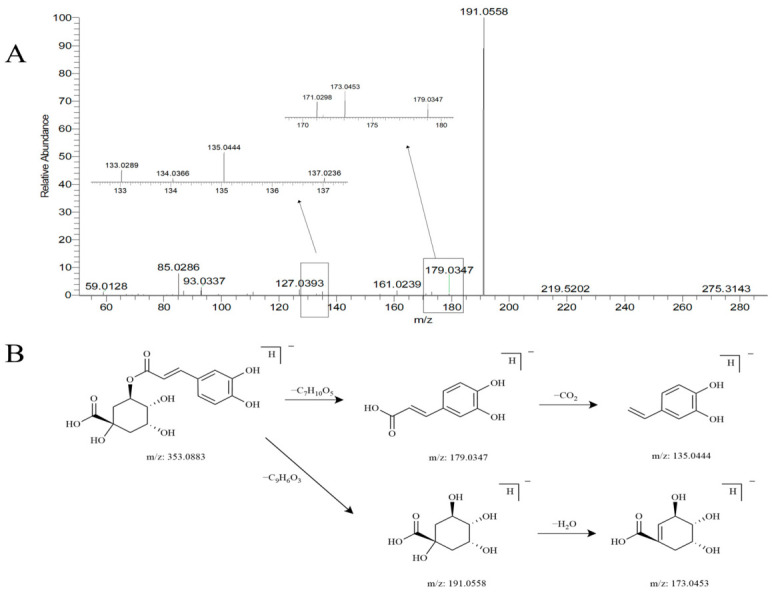
MS2 spectra (**A**) and fragmentation pathways (**B**) of chlorogenic acid.

Compound **26**, in negative ion mode, exhibited an excimer ion peak of m/z 353.0883 [M−H]^−^ in the first-stage mass spectrometry, corresponding to the molecular formula C_16_H_18_O_9_. Second-stage mass spectrometry analysis revealed that the parent ion [M−H]^−^ fragmented, losing C_9_H_6_O_3_ and C_7_H_10_O_5_ to produce two fragment ions, namely m/z 191.0558 [M-H-C_9_H_6_O_3_]^−^ and m/z 179.0347 [M-H-C_7_H_10_O_5_]^−^. Subsequently, the fragment ion at m/z 191.05559 lost one molecule of H_2_O to form the fragment ion m/z 173.0453 [M-H-C_9_H_6_O_3_-H_2_O]^−^, whilst another fragment ion at m/z 179.03392 further lost one molecule of CO_2_ to yield the fragment ion m/z 135.0444 [M-H-C_7_H_10_O_5_-CO_2_]^−^. Based on database searches, comparison with literature data and reference standards, compound **26** was identified as chlorogenic acid [47]. Its possible cleavage pattern is shown in Figure 9.

**Figure 10:**
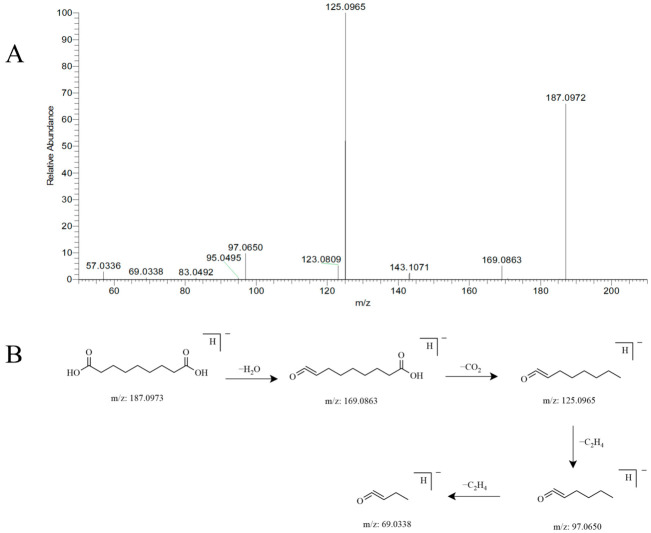
MS2 spectra (**A**) and fragmentation pathways (**B**) of azelaic acid.

During mass spectrometry fragmentation, fragment ions of compound **48** (m/z 187.0973 [M−H]^−^, C_9_H_16_O_4_) were detected at m/z 169.0863, m/z 125.0965, m/z 97.0650, and m/z 69.0338. Analysis revealed that the parent ion underwent a specific fragmentation reaction, whereby the parent ion first lost one molecule of H_2_O to produce the m/z 169.0863 [M-H-H_2_O]^−^, and subsequently this fragment ion undergoes further cleavage, sequentially losing CO_2_, C_2_H_4_ and C_4_H_8_ to yield m/z 125.0965 [M-H-H_2_O-CO_2_]−, m/z 97.0650 [M-H-H_2_O-CO_2_-C_2_H_4_]^−^, and m/z 69.0338 [M-H-H_2_O-CO_2_-C_4_H_8_]−. It is inferred that the compound is azelaic acid [28], and its possible cleavage pattern is shown in Figure 10.

### 2.5. Identification and Structural Analysis of Triterpenes

In this study, three triterpenoid compounds were identified from SMD, all of which were derived from Glycyrrhizae Radix et Rhizoma. Two triterpenoid compound nodes were identified in the MN: Licoricesaponin G2 (69) and glycyrrhizinic acid (73). As most triterpenes in Glycyrrhizae Radix et Rhizoma possess a glucuronic acid moiety at the R2 position, they typically exhibit the elimination of the glucuronic acid group (GlcA; C6H8O6, 176 Da) during high-energy collision-induced dissociation.

**Figure 11:**
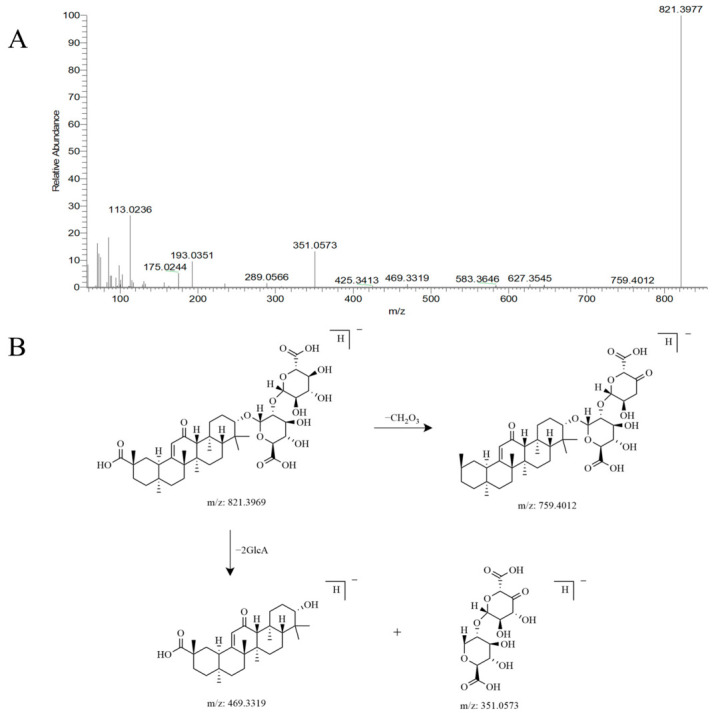
MS2 spectra (**A**) and fragmentation pathways (**B**) of glycyrrhizinic acid.

In negative ion mode, the quasi-molecular ion peak of compound 73 was detected at m/z 821.3969 [M−H]^−^, corresponding to the molecular formula C_42_H_62_O_16_, and produced the following secondary fragment ions: m/z 759.4012, m/z 469.3319, m/z 351.0573. The m/z 759.4012 [M-H-H_2_O-CO_2_]^−^ peak was obtained from the parent ion after the loss of one molecule of H_2_O and one molecule of CO_2_; m/z 469.3319 [M-H-2GlcA]^−^ peak and m/z 351.0573 [M-H-C_30_H_46_O_4_]^−^ fragment ions were obtained from the parent ion after the loss of two molecules of glucuronic acid (GlcA). Based on database searches, the literature [61] and comparison with reference standards, the compound was identified as glycyrrhizinic acid. Its possible cleavage pattern is shown in Figure 11.

### 2.6. Identification and Structural Analysis of Amino Acids

This study screened and identified four amino acid-derived compounds, including Histidine (1), Phenylalanine (34), Tryptophan (22), etc. The splitting rule of these amino acid-derived compounds primarily involves the carboxyl and amino functional groups, with α-cleavage being the main pathway, often accompanied by the loss of carboxyl and amino radicals.

**Figure 12:**
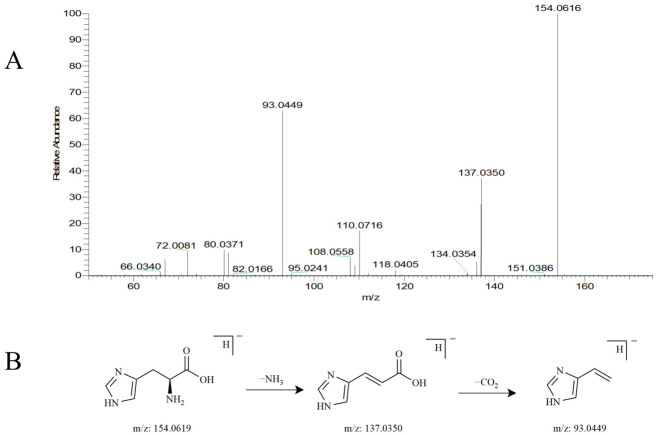
MS2 spectra (**A**) and fragmentation pathways (**B**) of histidine.

The precursor ion of compound **1** is m/z 154.0619 [M−H]^−^, and software fitting indicates the molecular formula as C_6_H_9_N_3_O_2_. Secondary mass spectrometry data show that the precursor ion lost an amino group to form m/z 137.0350 [M-H-NH_3_]^−^, followed by the loss of a carbonyl group to form m/z 93.0449 [M-H-NH_3_-CO_2_]^−^. Based on this secondary fragmentation data, combined with database and literature comparisons [25], compound **1** is proposed to be Histidine. Its possible cleavage pattern is shown in Figure 12.

The secondary mass spectrum of compound **35** (m/z 164.0712 [M−H]^−^, C_9_H_11_NO_2_) shows the following characteristic fragment ions: m/z 147.0445 [M-H-NH_3_]^−^, 120.0447 [M-H-CO_2_]^−^, and m/z 103.9194 [M-H-CO_2_-NH_3_]^−^. By comparison with relevant literature [55], it can be determined that compound **46** is Phenylalanine.

### 2.7. Identification and Structural Analysis of Alkaloids

Alkaloid compounds exhibit strong responses in positive ion mode, and α-cleavage readily occurs during mass spectrometric fragmentation, yielding corresponding characteristic fragment ions. In this study, two alkaloid compounds were identified from SMD. Compound **8** exhibited a parent ion peak at m/z 118.0962 [M+H]^+^ in positive ion mode. During fragmentation, α-cleavage occurred, yielding fragment ions m/z 59.0548 [M+H-N(CH_3_)_3_]^+^ and m/z 58.9149 [M+H-CH_3_COOH]^+^. Based on its cleavage pattern and characteristic fragment ions, and by cross-referencing with the database, reference standard and literature data [28,33], it was identified as betaine.

### 2.8. Identification and Structural Analysis of Other Constituents

In addition to the eight compounds mentioned above, other types of compounds, including coumarins, lignans, phenols, aldehydes, etc., were identified in the SMD. These were also confirmed by comparison with characteristic fragment ions reported in databases and existing literature. Taking Pteryxin (83) as an example, in positive ion mode, the parent ion peak is m/z 404.1702 [M+NH_4_]^+^, and the secondary mass spectrum reveals characteristic fragment ions including m/z 287.0915 [M+H-CH_3_CH=C(CH_3_)COOH]^+^ and m/z 245.0804 [M+H-CH_3_COO-CH3CH=C(CH_3_)CO]^+^, and m/z 227.0701 [M+H-CH_3_COOH-CH3CH=C(CH_3_)COOH]^+^. According to data from the literature, this is inferred to be pteryxin [85].

## 3. Discussion

In China, SMD, as a classic formula with extensive clinical applications throughout history, has attracted widespread attention from researchers and industry; however, the pharmacologically active constituents underlying its efficacy have not yet been fully elucidated. Currently, in the field of TCM analysis, liquid chromatography-tandem mass spectrometry (LC-MS/MS) plays a dominant role in the analysis of TCM compound formulations. However, the analysis and annotation of the vast amounts of mass spectrometry data generated by this technique represent a significant bottleneck in current research [88]. GNPS, as an online analytical platform, can cluster molecules with similar fragment ions into clusters and visualize them as molecular networks. By calculating fragment ion similarity, it reveals the chemical relationships between molecules, thereby enabling the rapid identification of complex chemical components [89].

By integrating high-resolution mass spectrometry with the GNPS platform, this study has established a research strategy suitable for the rapid analysis and structural identification of chemical constituents in TCM prescriptions, effectively addressing the high costs and low efficiency associated with traditional methods in the discovery of TCM constituents [90]. The identification results indicate that flavonoids, organic acids and sugars constitute the main constituents of SMD. Pharmacological studies indicate that these constituents generally exhibit anti-inflammatory, antitumor and antioxidant activities. For example, flavonoids, such as isoliquiritigenin, liquiritin, quercetin, etc., have been demonstrated to possess potential antitumor properties [91]. Quercetin has also shown positive effects in efficacy assessments in mouse models of asthma [92]. In a chronic bronchitis model, kaempferol and quercetin have been found to mediate the anti-inflammatory effects of SMD by reducing interleukin-6 levels; meanwhile, network pharmacology and molecular docking studies have revealed that both are key components in the treatment of radiation pneumonitis and chronic bronchitis [6,7]. Oxidative stress is a key factor in the development of chronic bronchitis [93]. The flavonoids identified in this study, such as rutin, can significantly reduce MDA levels in lung tissue from rats with pulmonary fibrosis, increase glutathione (GSH) levels and superoxide dismutase (SOD) activity in lung tissue, enhance total antioxidant capacity in serum, and reduce nitric oxide (NO) levels in lung tissue [94]. Furthermore, the homoisoflavonoids compounds methylophiopogonanone A and methylophiopogonanone B, derived from Ophiopogonis Radix, both possess good antioxidant activity [95,96]. An animal study demonstrated that Chlorogenic acid can enhance the antioxidant capacity of lung tissue, inhibit the spread of inflammation, and prevent paraquat-induced pulmonary fibrosis [97]. Polysaccharides derived from Ophiopogonis Radix and Polygonati Odorati Rhizoma have also been shown to improve inflammation and lung injury [98,99].

Terpenoids possess a variety of pharmacological activities, including anti-inflammatory, anti-tumor, antibacterial, antioxidant properties, etc. For example, 18-β-glycyrrhetinic acid not only exhibits anti-inflammatory effects [100], but in lung cancer research, it has also been shown to induce apoptosis in A549 cells, arrest the cell cycle, and inhibit cell migration. It is therefore considered a highly promising candidate for the treatment of lung cancer [101]. Licoricesaponin G2 can inhibit the activation of the TNF-α signaling pathway, modulate the epithelial–mesenchymal transition and remodeling of the extracellular matrix, thereby effectively alleviating the symptoms of pulmonary fibrosis [102]. In the field of anti-tumor therapy, it can also inhibit the PI3K/AKT signaling pathway, modulate the characteristics of tumor stem cells, and induce ferroptosis, thereby exerting an anti-lung cancer effect [103]. These compounds partially explain the pharmacological basis underlying SMD’s anti-inflammatory, anti-fibrotic, anti-tumor and antioxidant activities, which are particularly relevant to their application in the treatment of lung diseases. It is worth noting that the characteristic chemical constituents identified in this study via mass spectrometry not only align with the traditional uses of SMD in the treatment of pulmonary diseases, but also provide some evidence supporting a new application of SMD in alleviating cognitive impairment induced by chronic intermittent hypoxia (the neuroprotective effects of isoliquiritigenin) [3,104].

Furthermore, as research progresses, an increasing number of researchers are combining molecular networking with other mass spectrometry processing software to provide insights for the discovery of new compounds. Molecular networking is a technique developed on the basis of comparing MS/MS spectra; consequently, a high-quality MS/MS database is crucial for annotating the chemical composition of compounds in molecular networking analysis [89]. In the visualization map of this experiment, we can observe a large number of unassigned clusters and nodes. This suggests that many compounds cannot be effectively identified. Currently, the MS/MS fragment libraries available on the GNPS platform contain limited MS1 and MS/MS data regarding TCM herbs or their formulations. Future research should actively encourage the expansion of mass spectrometry databases related to traditional Chinese medicinal materials, whilst prioritizing the introduction of additional computational tools for compound identification to enhance the annotation rate of individual constituents.

In addition to characterizing the overall chemical composition of SMD, this study analyzed individual decoction samples of the seven TCM herbs comprising SMD, and attributed the chemical constituents identified in SMD to TCM herbs. This provides a reference for subsequent analyses of components migrating into the bloodstream and for research into the pharmacologically active constituents of the compound. The findings of this study are of significant importance for the clinical application of SMD, and the identification of multiple active constituents opens up possibilities for personalized medicine. However, this study still has certain limitations: firstly, although UHPLC-Q-Exactive Orbitrap-MS technology offers extremely high sensitivity and resolution for the qualitative analysis of compounds, it is unable to distinguish between stereoisomers directly. Given the complex composition of traditional Chinese medicine, which contains a large number of isomers, the identification of these isomers is a highly challenging task that still requires the use of specialized techniques such as chiral columns and ion mobility mass spectrometry [105].

Secondly, the quality of TCM formulations is fundamental to ensuring consistent therapeutic efficacy; therefore, quantitative analysis is essential. The quality of TCM formulations depends directly on the content of their active ingredients; quality control must therefore involve quantitative analysis of the bioactive substances to determine the specific content and contribution of each compound within the formulation. Although existing research has already made it possible to make preliminary predictions regarding the active components in SMD, further in-depth pharmacological experiments are required to verify the specific active substances, confirm the quality markers of SMD, and subsequently establish a comprehensive quality control system for these markers [106]. In the next phase of this study, we will further combine major classes of compounds or individual active substances associated with the primary pharmacological effects of SMD to establish quantitative analytical methods for the major constituents of SMD. This will ensure the reliability of SMD in modern clinical applications.

## 4. Materials and Methods

### 4.1. Medicines and Reagents

The SMD freeze-dried powder (251209) and the freeze-dried powders obtained by decocting the seven TCM herbs separately (251109–251115) were both supplied by Sichuan Pu Hua TCM Technology Co., Ltd (Chengdu, China). The 14 reference standards include rutin (PS012233), quercetin (PS014278), glycyrrhizin (batch no.: PS012028), glycyrrhizinic acid (PS021263), chlorogenic acid (PS014337), isoliquiritigenin (PS021101), caffeic acid (PS014204), methylophiopogonanone A (PS011641), methylophiopogonanone B (PS000488), hyperoside (PS014172), formononetin (PS000674), kaempferol (PS012233), apigenin (PS013992) and betaine (PS012048), all purchased from Chengdu Push Bio-Technology Co., Ltd. (Chengdu, China)

Analytical-grade chemicals included: acetonitrile and methanol (HPLC grade, Thermo Fisher Scientific, Chengdu, China); formic acid (LC-MS grade, Thermo Fisher Scientific, Chengdu, China); ultrapure distilled water (Watson’s Water, Hong Kong, China).

### 4.2. Sample Preparation for UHPLC-Q Exactive Orbitrap HRMS Analysis

Approximately 0.5 g of SMD freeze-dried powder and 0.5 g of freeze-dried powder from the decoction of each individual TCM herb were accurately weighed, placed in stoppered conical flasks, and 50 mL of 50% (*v*/*v*) methanol was carefully added. The flasks were tightly sealed, weighed, subjected to ultrasonic treatment (power 250 W, frequency 40 kHz) for 30 min, allowed to cool, weighed again, made up to the weight lost during the ultrasonic extraction with 50% methanol, shaken well, filter through a 0.22 µm polypropylene membrane filter.

### 4.3. Preparation of Standard Solutions

The 14 reference standards were precisely weighed, dissolved in 50% methanol and made up to volume to prepare a mixed solution containing 100 μg/mL of each compound. This solution was stored at 4 °C for subsequent UHPLC-MS analysis.

### 4.4. UHPLC-Q Exactive Orbitrap HRMS Analysis

UHPLC-HRMS analysis was performed on a Vanquish rapid separation binary system coupled with a Q Exactive Orbitrap mass spectrometer equipped with a heated electrospray ionization source (HESI), which was operated in both positive and negative ion modes (Thermo Fisher Scientific Inc., Waltham, MA, USA).

Chromatographic separation was performed using a Vanquish UHPLC system equipped with a binary pump and a Waters ACQUTIY UPLC ^®^ HSS T3 column (100 × 2.1 mm, 1.9 μm particle size; Waters Corporation, Chengdu, China) maintained at constant temperature (30 °C). The mobile phase consisted of acetonitrile (phase A) and ultrapure water containing 0.1% (*v*/*v*) formic acid (phase B) for both positive and negative ionization modes. The flow rate was maintained at 0.30 mL/min with an injection volume of 2 μL. The optimized elution gradient was as follows: 0–1 min, 5% A; 1–2 min, 5–7.5% A; 2–4 min, 7.5–11% A; 4–7 min, 11–14% A; 7–8 min, 14–15% A; 8–18 min, 15% A; 18–26 min, 15–40% A; 26–30 min, 40–95% A; 30–34 min, 95% A; 34–35 min, 95–5% A; 35–36 min, 5% A.

Mass spectrometry analysis was performed using a Q-Exactive Orbitrap MS, with detection in both positive and negative ion modes via the HESI source. The ion spray voltages were set at 3.5 kV (+) and 3.2 kV (−), respectively. The sheath gas flow rate was set to 35.0 Arb, and the carrier gas flow rate to 10 Arb. The probe heater temperature was 350 °C (+) and 300 °C (−). The scanning mode was set to Full MS/data-dependent MS2 (Full MS/dd-MS2). The primary Full MS selected a resolution of 70000 FWHM, with the dd-HRMS2 resolution set to 17,500 and the scan range of 100–1500. The stepped normalized collision energies (NCEs) were 20, 40, and 60 eV. S-lens RF level was 50.

### 4.5. Data Processing and Analysis Strategy

Firstly, a systematic search was conducted in PubMed, Web of Science, the Wanfang databases and China National Knowledge Infrastructure (CNKI) for literature on the chemical constituents of the seven TCM herbs comprising SMD. Information such as chemical formulas, molecular weights and secondary fragment ions was collated to establish a database of SMD’s chemical constituents.

Next, the raw data acquired by high-resolution mass spectrometry were pre-processed using the Compound Discover 3.3 mass spectrometry data processing software to establish a workflow for the identification of unknown compounds. The Xcalibur 4.0 workstation was used to calculate the exact molecular masses of the unknown compounds and to extract information such as protonated ion peaks and characteristic fragment ions.

Initially, raw mass spectrometry data were matched and screened against the SMD chemical composition database and the ChemSpider database; subsequently, unknown chemical components were identified by cross-referencing secondary mass spectrometry fragments with mzCloud, the mzVault secondary mass spectrometry database, the HMDB (https://hmdb.ca/, accessed on 5 February 2026) database, reference standards, and our own SMD database (whilst simultaneously comparing the mass spectrometry data with blank samples to further rule out interference). In addition, we compared the SMD mass spectrometry data with that obtained from individual TCM herbs to identify the sources of the chemical constituents in the SMD. Thirdly, using GNPS molecular network technology, compounds with similar MS/MS fragmentation patterns are grouped into molecular clusters connected by multiple nodes, producing visualized results that enable the detection and identification of unknown compounds based on fragment similarity. The workflow for constructing and analyzing GNPS molecular networks is as follows: Using ProteoWizard MSconvert software (version 3.0.26070), the raw mass spectrometry data files were converted into mzML format and uploaded via FileZilla to GNPS for data analysis (https://gnps.ucsd.edu, accessed on 15 March 2026), resulting in the generation of a molecular network.

During the construction of the molecular network, the cosine score threshold was set to 0.7, with a mass tolerance of 0.02 Da for both precursor and fragment ions, and a minimum of 6 matching fragment ions; all other parameters were left at their default values. Finally, the generated molecular network was visualized using Cytoscape software (version 3.10.0). Based on the fragment ion information provided by the GNPS platform and database, and in conjunction with fragmentation patterns reported in the literature, the identified components were compared and verified. Fragmentation patterns were summarized, and the chemical composition of SMD was determined and analyzed.

## 5. Conclusions

This study is the first to comprehensively identify the constituents of SMD using UHPLC-Q Exactive orbitrap HRMS combined with MN, identifying a total of 86 chemical components (including flavonoids, homoisoflavonoids, organic acids, alkaloids, amino acids, saccharides, triterpenes and other constituents). Furthermore, by analyzing decoction samples of individual herbs of SMD, this study identified the TCM herbal origins of the chemical constituents in SMD. This study lays the chemical foundation for the subsequent screening of bioactive compounds and quality control of SMD, whilst providing a reliable scientific basis for elucidating their pharmacological mechanisms. It also offers an effective method for the rapid qualitative analysis of chemical constituents in TCM prescriptions.

## Figures and Tables

**Table 1:** Identification of chemical components in SMD by UHPLC-Q Exactive orbitrap HRMS.

NO	Compound Name	RT (min)	Formula	Ion Mode	Theoretical Mass m/z	Experimental Mass m/z	Error (ppm)	Major Ion Fragments MS/MS (m/z)	Compound Type	Source	Refs.
1	Histidine	0.83	C_6_H_9_N_3_O_2_	[M−H]^−^	154.0619	154.0622	−1.95	154.0616, 137.0350, 110.0716, 95.0241, 93.0449	Amino acids	SS, MD, YZ, THF, BBD, GC	[25]
2	Iditol	0.86	C_6_H_14_O_6_	[M−H]^−^	181.0714	181.0717	−1.66	101.0236, 89.0235, 71.0129	Saccharides	SS, YZ, THF, BBD, GC, SY	[26]
3	Sucrose	0.89	C_12_H_22_O_11_	[M−H]^−^	341.109	341.1089	0.29	179.0710, 119.0495, 89.0238	Saccharides	SS, MD, YZ, BBD, GC, SY	[27]
4	α,α-Trehalose	0.90	C_12_H_22_O_11_	[M−H]^−^	341.109	341.1089	0.29	341.1093, 179.0557, 119.0557, 101.0236, 89.0235	Saccharides	SS, MD, YZ, THF, BBD, GC, SY	[28,29]
5	Quinic acid	0.92	C_7_H_12_O_6_	[M−H]^−^	191.0557	191.0561	−2.09	191.0557, 173.0086, 127.0394	Organic acids	SS, BBD, SY	[28]
6	Isocitric acid	0.97	C_6_H_8_O_7_	[M−H]^−^	191.0194	191.0197	−1.57	191.0557, 111.0079, 85.0285, 71.0128	Organic acids	SS, MD, YZ, THF, BBD, GC, SY	[30]
7	Fructose	1.04	C_6_H_12_O_6_	[M−H]^−^	179.0557	179.0561	−2.23	119.034, 113.0236, 101.0239, 89.0235	Saccharides	SS, MD, YZ, THF, BBD, GC, SY	[31]
8	Galactose	1.05	C_6_H_12_O_6_	[M−H]^−^	179.0557	179.0561	−2.23	89.0235, 71.0128, 85.0284, 59.0128	Saccharides	SS, MD, YZ, THF, BBD, GC, SY	[32]
9 *	Betaine	1.26	C_5_H_11_NO_2_	[M+H]^+^	118.0962	118.0963	−0.85	59.0785, 58.9149	Alkaloids	SS, MD, YZ, THF, BBD, SY	[28,33]
10	trans-Aconitic acid	1.43	C_6_H_6_O_6_	[M−H]^−^	173.0087	173.0091	−2.31	129.0186, 111.0079, 85.0285	Organic acids	SS, MD, YZ, THF, BBD, GC, SY	[34]
11	3-Butene-1,2,3-tricarboxylic acid	1.63	C_7_H_8_O_6_	[M−H]^−^	187.0246	187.0248	−1.07	143.0343, 125.0969, 115.0393	Organic acids	SS, BBD	[35]
12	3-Hydroxy-3-(methoxycarbonyl)pentanedioic acid	2.53	C_7_H_10_O_7_	[M−H]^−^	205.0354	205.0353	0.49	173.0083, 129.0186, 111.0079	Organic acids	SS	[36]
13	Glutaric acid	2.58	C_5_H_8_O_4_	[M−H]^−^	131.0344	131.0349	−3.82	87.0443, 69.0336, 59.0127	Organic acids	THF, BBD, GC, SY	[37]
14	Thymidine	3.06	C_10_H_14_N_2_O_5_	[M−H]^−^	241.0832	241.0829	1.24	241.0831, 151.0508, 125.0340	Others	THF, SY	[38]
15	Thymine	3.12	C_5_H_6_N_2_O_2_	[M+H]^+^	127.0497	127.0502	−3.94	127.0495, 110.0329, 84.0518	Alkaloids	SS, MD, YZ, THF, BBD, GC, SY	[38]
16	Methylsuccinic acid	3.54	C_5_H_8_O_4_	[M−H]^−^	131.0344	131.0349	−3.82	131.0342, 101.0235, 87.0442, 85.0285	Organic acids	THF, BBD, GC, SY	[39]
17	4-Methoxysalicylic acid	3.795	C_8_H_8_O_4_	[M−H]^−^	167.0346	167.0349	−1.80	123.0443, 108.0208	Organic acids	SY	[40]
18	5-Aminovaleric acid	4.05	C_5_H_11_NO_2_	[M−H]^−^	116.0712	116.0717	−4.31	117.0549, 99.00784, 59.0127	Organic acids	THF, BBD, GC, SY	[41]
19	(1,3-Phenylenedioxy)diacetic acid	4.43	C_10_H_10_O_6_	[M−H]^−^	225.0408	225.0404	1.78	135.0443, 121.0287, 59.0128	Organic acids	GC	[42]
20	3,5-Dihydroxybenzoic acid	4.43	C_7_H_6_O_4_	[M−H]^−^	153.0189	153.0193	−2.61	153.0187, 109.0286	Organic acids	SS, THF, BBD, GC, SY	[43]
21	Adipic acid	4.67	C_6_H_10_O_4_	[M−H]^−^	145.0502	145.0506	−2.76	101.0599, 83.0493	Organic acids	THF, GC, SY	[44]
22	Tryptophan	5.28	C_11_H_12_N_2_O_2_	[M−H]^−^	203.0825	203.0826	−0.49	142.0656, 116.0498	Amino acids	SS, MD, YZ, THF, GC, SY	[45]
23	Xanthine	5.38	C_5_H_4_N_4_O_2_	[M−H]^−^	151.0265	151.0261	2.65	108.0209, 71.0129	Others	THF, GC	[46]
24	Neochlorogenic acid	5.52	C_16_H_18_O_9_	[M−H]^−^	353.0886	353.0878	2.27	353.0876, 191.0553, 179034, 135.0445	Organic acids	SY	[47]
25	Salicylic acid	5.97	C_7_H_6_O_3_	[M−H]^−^	137.0238	137.0244	−4.38	109.0288, 93.0337	Organic acids	MD, YZ, THF, GC, SY	[28]
26 *	Chlorogenic acid	6.76	C_16_H_18_O_9_	[M−H]^−^	353.0883	353.0878	1.42	191.0558, 179.0347, 173.0453, 135.0444	Organic acids	SY	[47]
27	4-Anisic acid	7.23	C_8_H_8_O_3_	[M−H]^−^	151.0395	151.0401	−3.97	107.0498, 109.0287	Organic acids	GC, SY	[48,49]
28	Pimelic acid	7.32	C_7_H_12_O_4_	[M−H]^−^	159.0657	159.0663	−3.77	115.0756, 97.0650	Organic acids	THF, GC	[44]
29 *	Caffeic acid	7.87	C_9_H_8_O_4_	[M−H]^−^	179.0348	179.035	−1.12	135.0445, 107.0492	Organic acids	MD, GC, SY	[50,51]
30	6-Hydroxycaproic acid	7.89	C_6_H_12_O_3_	[M−H]^−^	131.0707	131.0713	−4.58	85.0649, 59.0127	Organic acids	MD, THF, BBD, GC, SY	[52]
31	Gentisic acid	8.05	C_7_H_6_O_4_	[M−H]^−^	153.0188	153.0193	−3.27	153.0187, 109.0286, 91.0180	Organic acids	SS, MD, YZ, THF, BBD, GC, SY	[28,53]
32	N-Acetyl-D-alloisoleucine	8.55	C_8_H_15_NO_3_	[M−H]^−^	172.0975	172.0979	−2.32	130.0867, 172.0974, 58.0286	Amino acids	YZ, THF, BBD, GC, SY	[31]
33	3-Methylhistamine	8.56	C_6_H_11_N_3_	[M+H]^+^	126.1019	126.1025	−4.76	126.1017, 108.0535, 96.0605	Others	SY	[32]
34	Phenylalanine	9.46	C_9_H_11_NO_2_	[M−H]^−^	164.0712	164.0717	−3.05	147.0444, 120.0447, 103.9194	Amino acids	SS, MD, YZ, THF, BBD, GC, SY	[54]
35	3-Phenyllactic acid	10.50	C_9_H_10_O_3_	[M−H]^−^	165.0553	165.0557	−2.42	165.0552, 147.0444, 119.0494	Organic acids	SY	[55]
36	2-Hydroxycinnamic acid	10.84	C_9_H_8_O_3_	[M−H]^−^	163.0396	163.0401	−3.07	163.0395, 119.0494, 93.0337	Organic acids	SS, MD, YZ, THF, BBD, GC, SY	[56]
37	Corymboside	11.33	C_26_H_28_O_14_	[M+H]^+^	565.1556	565.1552	0.71	427.1022, 409.0915, 379.0779, 325.0710, 295.0603, 203.0338	Flavonoids	GC	[57]
38	Schaftoside	11.45	C_26_H_28_O_14_	[M+H]^+^	565.1558	565.1552	1.06	547.1461, 529.1334, 445.1071, 355.0822	Flavonoids	GC	[58]
39	5,7-Dihydroxy-4-methylcoumarin	12.26	C_10_H_8_O_4_	[M+H]^+^	193.0497	193.0495	1.04	178.0301, 165.0603, 149.0683	Coumarins	BBD, SY	[59]
40	Daidzin	12.74	C_21_H_20_O_9_	[M+H]^+^	417.1184	417.118	0.96	255.0651, 227.0706, 137.0347	Flavonoids	GC	[60]
41 *	Liquiritin	14.25	C_21_H_22_O_9_	[M−H]^−^	417.1193	417.1191	0.48	255.0665, 135.0081, 119.0495	Flavonoids	GC, SY	[61,62]
42	4-Indolecarbaldehyde	14.78	C_9_H_7_NO	[M−H]^−^	144.0448	144.0455	−4.86	116.14944	Aldehydes	THF	[42]
43	Genistin	15.75	C_21_H_20_O_10_	[M+H]^+^	433.1135	433.1129	1.39	413.1137, 271.0600	Flavonoids	GC	[60]
44 *	Rutin	15.80	C_27_H_30_O_16_	[M−H]^−^	609.1473	609.1461	1.97	300.02777, 271.0249, 178.9975, 151.0026	Flavonoids	SY	[60]
45 *	Hyperoside	15.99	C_21_H_20_O_12_	[M+H]^+^	465.1036	465.1028	1.72	303.0498, 153.0273, 85.0359	Flavonoids	SY	[63]
46 *	Quercetin	16.09	C_15_H_10_O_7_	[M+H]^+^	303.0500	303.0499	0.33	303.0498, 285.0387, 257.0441, 165.0238	Flavonoids	GC, SY	[47,60]
47	4-Nitrophenol	17.11	C_6_H_5_NO_3_	[M−H]^−^	138.0191	138.0197	−4.35	138.0189, 108.0208	Phenols	THF, GC, SY	[64]
48	Azelaic acid	21.01	C_9_H_16_O_4_	[M−H]^−^	187.0973	187.0976	−1.60	169.0863, 125.0965, 97.0650, 69.0338	Organic acids	SS, MD, YZ, THF, BBD, GC, SY	[28]
49	Kuromanin	21.87	C_21_H_20_O_11_	[M+H]^+^	449.1081	449.1078	0.67	287.0547, 258.0519, 241.0691	Flavonoids	THF, BBD, GC, SY	[65]
50	Fisetin	21.87	C_15_H_10_O_6_	[M+H]^+^	287.0552	287.055	0.70	241.0502, 213.0549	Flavonoids	SY	[66]
51	Isokaempferide	21.93	C_16_H_12_O_6_	[M−H]^−^	299.0564	299.0561	1.00	284.0328, 173.0233	Flavonoids	GC	[67]
52	Apigetrin	22.21	C_21_H_20_O_10_	[M−H]^−^	431.0992	431.0983	2.09	268.0381, 269.0456, 151.0032	Flavonoids	GC	[28,68]
53	Isochlorogenic acid C	22.89	C_25_H_24_O_12_	[M−H]^−^	515.1204	515.1195	1.75	353.0881, 191.0558, 179.0345, 135.0443	Organic acids	SY	[69]
54	Neohesperidin	22.95	C_28_H_34_O_15_	[M−H]^−^	609.1841	609.1825	2.63	609.1838, 301.0721	Flavonoids	YZ, BBD, SY	[28,70]
55	Glycitin	23.04	C_22_H_22_O_10_	[M+H]^+^	447.129	447.1286	0.89	285.0756, 270.0522	Flavonoids	GC	[50]
56	Ononin	23.49	C_22_H_22_O_9_	[M+H]^+^	431.134	431.1337	0.70	267.0692, 254.0806, 137.0347	Flavonoids	GC	[71]
57	Isoliquiritin	23.75	C_21_H_22_O_9_	[M+H]^+^	419.1344	419.1337	1.67	257.0806, 163.0468	Flavonoids	GC	[62]
58	Daidzein	23.91	C_15_H_10_ O_4_	[M+H]^+^	255.0654	255.0653	0.39	255.0650, 199.0754, 181.0698, 137.0345	Flavonoids	GC	[60]
59	5,7-dihydroxy-2-phenyl-4H-chromen-4-one	23.92	C_15_H_10_O_4_	[M+H]^+^	225.0654	255.0652	0.89	213.0556, 153.0786	Flavonoids	GC	[72]
60	Luteolin	24.70	C_15_H_10_O_6_	[M−H]^−^	285.041	285.0405	1.75	257.0459, 241.0499, 151.0034, 133.0288	Flavonoids	GC	[60]
61	Emodin	24.79	C_15_H_10_O_5_	[M+H]^+^	271.0598	271.0601	−1.11	271.0961, 163.0460, 137.0709	Others	GC	[73]
62	Berberine	24.93	C_20_H_17_NO_4_	[M+H]^+^	336.1233	336.123	0.89	320.0915, 278.0810	Flavonoids	SS, MD, YZ, THF, BBD, GC, SY	[74]
63	Scrophulein	25.58	C_17_H_14_O_6_	[M+H]^+^	315.0863	315.0863	0.00	315.0861, 255.0650	Flavonoids	GC	[75]
64	3-Hydroxyanthranilic acid	25.76	C_7_H_7_NO_3_	[M−H]^−^	152.0346	152.0353	−4.60	152.0346, 122.0366	Organic acids	THF, SY	[76]
65	Corchorifatty acid F	26.51	C_18_H_32_O_5_	[M−H]^−^	327.2181	327.2177	1.22	229.1444, 211.1337, 171.1021	Organic acids	SS, MD, YZ, THF, BBD, GC, SY	[27]
66 *	Apigenin	26.68	C_15_H_10_O_5_	[M+H]^+^	271.0607	271.0601	2.21	271.0963, 119.0591	Flavonoids	GC	[28]
67 *	Kaempferol	26.97	C_15_H_10_O_6_	[M−H]^−^	285.041	285.0405	1.75	285.0771	Flavonoids	GC	[28]
68	(15Z)-9,12,13-Trihydroxy-15-octadecenoic acid	27.44	C_18_H_34_O_5_	[M−H]^−^	329.2335	329.2334	0.30	229.1446, 211.1337, 171.1021, 139.1121, 99.0806	Organic acids	SS, MD, YZ, THF, BBD, GC	[44]
69	Licoricesaponin G2	27.57	C_42_H_62_O_17_	[M+H]^+^	839.408	839.406	2.38	469.3310, 451.3205	Triterpenes	GC	[62]
70	Diosmetin	27.67	C_16_H_12_O_6_	[M+H]^+^	301.0705	301.0707	−0.66	286.0471, 258.0523, 153.0274	Flavonoids	GC	[28]
71 *	Isoliquiritigenin	27.99	C_15_H_12_O_4_	[M−H]^−^	255.0668	255.0663	1.96	255.0666, 153.0188, 135.0081, 119.0495, 91.0180	Flavonoids	GC	[61,77]
72	Dodecanedioic acid	28.05	C_12_H_22_O_4_	[M−H]^−^	229.1447	229.1445	0.87	229.1445, 211.1337, 167.1435	Organic acids	SS, THF, SY	[38]
73 *	Glycyrrhizinic acid	28.07	C_42_H_62_O_16_	[M−H]^−^	821.3969	821.3965	0.49	759.4012, 469.3319, 351.0573	Triterpenes	GC	[61]
74	18-β-Glycyrrhetinic acid	28.09	C_30_H_46_O_4_	[M+H]^+^	471.3463	471.3469	−1.27	453.3360, 435.3269, 425.3409, 407.3297, 317.2117, 235.1696, 135.1278	Triterpenes	GC	[78]
75 *	Formononetin	28.14	C_16_H_12_O_4_	[M−H]^−^	267.0668	267.0663	1.87	252.0429, 223.0399, 195.0450	Flavonoids	BBD, GC	[28,79]
76	9-HpODE	28.86	C_18_H_32_O_4_	[M−H]^−^	311.223	311.2228	0.64	293.2128, 267.1968, 223.2064	Organic acids	SS, MD, YZ, THF, BBD, GC, SY	[80]
77	Nobiletin	28.92	C_21_H_22_O_8_	[M+H]^+^	403.1391	403.1387	0.99	403.1388, 373.0918, 327.0858, 211.0234, 183.0311	Flavonoids	SS, MD, YZ, BBD, GC	[74,81]
78	Sesamin	29.23	C_20_H_18_O_6_	[M+H]^+^	355.1169	355.1176	−1.97	337.0998, 135.0552	Lignans	GC	[50]
79	12(13)-DiHOME	29.35	C_18_H_34_O_4_	[M+H]^+^	315.2532	315.253	0.63	243.0650, 201.0542	Organic acids	GC	[82]
80 *	Methylophiopogonanone A	30.15	C_19_H_18_O_6_	[M+H]^+^	343.1182	343.1176	1.75	207.0655, 189.0575, 135.0553	Homoisoflavonoids	MD	[83]
81 *	Methylophiopogonanone B	30.18	C_19_H_20_O_5_	[M+H]^+^	327.1233	327.1238	−1.53	207.0653, 121.0747, 107.0583, 219.0654, 237.0758	Homoisoflavonoids	MD	[83]
82	12-oxo Phytodienoic Acid	30.21	C_18_H_28_O_3_	[M+H]^+^	293.2106	293.2111	−1.71	293.2109, 275.2005, 219.1743, 121.1111, 107.0946	Organic acids	YZ, THF, GC, SY	[26,84]
83	Pteryxin	30.39	C_21_H_22_O_7_	[M+NH_4_]^+^	404.1702	404.1704	−0.49	287.0915, 245.0804, 227.0701	Coumarins	GC	[85]
84	13-HODE	30.53	C_18_H_32_O_3_	[M−H]^−^	295.2281	295.2279	0.68	277.2175, 295.2281, 195.1386	Organic acids	SS, MD, YZ, THF, BBD, GC, SY	[41]
85	3-Hydroxy myristic acid	30.65	C_14_H_28_O_3_	[M−H]^−^	243.1966	243.1966	0.00	243.1968, 59.0128	Organic acids	SS, YZ, GC, SY	[49]
86	α-Linolenic acid	31.73	C_18_H_30_O_2_	[M−H]^−^	277.2175	277.2173	0.72	277.1447, 121.0287, 91.0179	Organic acids	SS, MD, YZ, THF, BBD, SY	[86]

Note: “*” indicates that the compound was compared with the reference standard; “SS”, “MD”, “YZ”, “THF”, “BBD”, “GC” and ‘SY’ all refer to the medicinal herbs in the SMD, where “SS” is Adenophorae Radix, “MD” is Ophiopogonis Radix, “YZ” is Polygonati Odorati Rhizoma, “THF” is Trichosanthis Radix, “BBD” is Lablab Semen Album, “GC” is Glycyrrhizae Radix et Rhizoma, and “SY” is Mori Folium.

## Data Availability

The original contributions presented in this study are included in the article. Further inquiries can be directed to the corresponding authors.

## Abbreviations

The following abbreviations are used in this manuscript:

GNPS	Global Natural Products Social Molecular Networking
GSH	Glutathione
LC-MS	Liquid chromatography-mass spectrometry
MN	Molecular networking
NO	Nitric oxide
SMD	Shashen Maidong Decoction
SOD	Superoxide dismutase
TCM	Traditional Chinese Medicine
